# Low-Density Lipoprotein Cholesterol Target Attainment in Lithuania: A Nationwide Analysis of Real-World Health Data

**DOI:** 10.3390/medicina61081484

**Published:** 2025-08-19

**Authors:** Gediminas Urbonas, Tomas Lapinskas, Indrė Čeponienė, Olga Vasiliauskienė, Jelena Umbrasienė, Ingrida Grabauskytė, Jurgita Plisienė

**Affiliations:** 1 Department of Family Medicine, Medical Academy, Lithuanian University of Health Sciences, Eivenių 2, 50161 Kaunas, Lithuania; olga.vasiliauskiene@lsmu.lt; 2 Department of Cardiology, Medical Academy, Lithuanian University of Health Sciences, Eivenių 2, 50161 Kaunas, Lithuania; tomas.lapinskas@lsmu.lt (T.L.); indre.ceponiene@lsmu.lt (I.Č.); jurgita.plisiene@lsmu.lt (J.P.); 3 Lithuanian Society of Cardiology, Eivenių 2, 50161 Kaunas, Lithuania; 4 Department of Clinical Research, Saules Seimos Medicinos Centras, Partizanų 27D, 49449 Kaunas, Lithuania; j.umbrasiene@ssmc.lt; 5 Department of Physics, Mathematics and Biophysics, Lithuanian University of Health Sciences, Eivenių 4, 50103 Kaunas, Lithuania; ingrida.grabauskyte@lsmu.lt

**Keywords:** low-density lipoprotein cholesterol, lipid-lowering therapy, electronic health records, statins, ezetimibe

## Abstract

*Background and Objectives*: Low-density lipoprotein cholesterol (LDL-C) reduction is critical for cardiovascular disease (CVD) prevention. This study aimed to assess the proportion of patients achieving the LDL-C target in Lithuania and to identify factors associated with target achievement. *Materials and Methods*: This retrospective study used anonymized health data from the Electronic Health Services and Cooperation Infrastructure Information System (ESPBI IS) in Lithuania. Adults aged ≥40 years with at least one LDL-C measurement in 2023 and no documented cancer diagnosis were included. The primary outcome was the proportion of patients achieving LDL-C < 1.8 mmol/L, the target recommended by the European Society of Cardiology guidelines for high-risk individuals. Univariate logistic regression analysis was conducted to identify factors associated with achieving the LDL-C target. *Results*: The study included 396,835 patients (mean age, 66.9 years). The mean LDL-C concentration was 3.32 mmol/L, and only 8.1% of patients achieved LDL-C < 1.8 mmol/L. Target achievement was higher among patients in the secondary CVD prevention group compared to primary prevention (20.6% vs. 7.3%). Over half of patients (56.4%) received no lipid-lowering therapy (LLT). Statin monotherapy was the most prescribed LLT (31.3%), while only 2.7% of patients received statin and ezetimibe combination. In logistic regression analysis, secondary prevention status, more frequent cardiologist consultations, and higher LLT prescription frequency were associated with LDL-C target achievement. Compared to patients not receiving LLT, the odds of achieving LDL-C < 1.8 mmol/L were significantly higher in those receiving statin monotherapy (odds ratio [OR]: 3.153, 95% confidence interval [CI]: 3.069–3.240), statin and ezetimibe (OR: 7.631, 95% CI: 7.267–8.013), or statin and antihypertensive (OR: 3.945, 95% CI: 3.803–4.092). *Conclusions*: LDL-C target attainment remains low in Lithuania, with the underuse of LLT. Broader implementation of guideline-recommended lipid-lowering strategies is needed to improve LDL-C control.

## 1. Introduction

With atherosclerotic cardiovascular disease (CVD) continuing to be a major global health issue [1], its primary and secondary prevention remain key priorities in public health strategies aimed at reducing cardiovascular morbidity, mortality, and healthcare burden [2].

Low-density lipoprotein cholesterol (LDL-C) is a modifiable risk factor contributing to the onset and progression of atherosclerotic CVD [3]. Evidence from clinical trials and meta-analyses consistently demonstrates that lowering LDL-C reduces the risk of cardiovascular events [4,5,6]. Lowering LDL-C early, intensively, and consistently is fundamental for both primary and secondary atherosclerotic CVD prevention [7,8].

Recognizing the contribution of LDL-C levels in the development of atherosclerotic CVD, the current European Society of Cardiology/European Atherosclerosis Society (ESC/EAS) and ESC guidelines recommend LDL-C goals tailored to individual cardiovascular risk, i.e., reduction of at least 50% from baseline LDL-C levels, with absolute targets set at <1.4 mmol/L for individuals at very high cardiovascular risk, <1.8 mmol/L for those at high risk, <2.6 mmol/L for those at moderate risk, and <3.0 mmol/L for those at low risk [2,9]. These goals are to be achieved through a combination of lifestyle interventions and pharmacological treatment, beginning with high-intensity statins and, if necessary, adding ezetimibe or a proprotein convertase subtilisin/kexin type 9 (PCSK9) inhibitor [2,9].

Extensive evidence supports the use of statins at the highest tolerable dose, and their benefit strongly correlates with the degree of LDL-C reduction [10]. Statins reduce LDL-C levels and lower the risk of major cardiovascular events, including myocardial infarction, stroke, and cardiovascular death in both primary and secondary CVD prevention populations [11,12,13,14]. Combination therapy with ezetimibe offers an alternative strategy, particularly for patients who do not tolerate statins or fail to reach LDL-C targets with statins alone. Ezetimibe added to moderate-intensity statins has been shown to improve LDL-C levels and adherence, with fewer adverse effects compared to high-intensity statin monotherapy [15,16].

Despite clear guideline recommendations and the proven efficacy of statins, the use of high-dose statins, LDL-C goals achievement, and thus control of cardiovascular risk remains suboptimal [10,15]. Many patients do not achieve recommended LDL-C goals due to underuse of lipid-lowering therapy (LLT), including high-intensity statins and statin combination with ezetimibe, adverse effects, or poor adherence [10,17].

There is a lack of published data evaluating LDL-C control at the population level in Lithuania. Understanding current patterns of LDL-C goal attainment and associated factors is essential to identify potential gaps in care and guide future healthcare improvement efforts. The aim of this study was to evaluate the proportion of adult patients achieving the LDL-C target in Lithuania using national e-health data. Additionally, we aimed to identify factors associated with LDL-C target attainment. The findings of this study may have both clinical and public health implications, providing evidence to support targeted interventions for better LDL-C management, suggesting updates to national CVD prevention strategies or clinical practice guidelines, and advocating for reviewing reimbursement policies to ensure wider access to effective LLTs.

## 2. Materials and Methods

This was a retrospective analysis of national health data, extracted from the original version Electronic Health Services and Cooperation Infrastructure Information System (ESPBI IS) and provided by the Lithuanian State Data Agency (SDA), Vilnius, Lithuania. ESPBI IS is a digital platform used by healthcare institutions in Lithuania to manage patient health information. It contains comprehensive electronic health records, including diagnoses, referrals, results of laboratory tests, medical imaging, prescribed and dispensed medicines, and other medical information.

For this study, all data extracted from the ESPBI IS were fully anonymized prior to analysis by the data provider SDA, with all personal identifiers removed to ensure patient confidentiality. The study protocol was reviewed and approved by the Kaunas Regional Biomedical Research Ethics Committee (protocol code BE-2-121, issued on 18 December 2024). As the study involved secondary use of anonymized data, informed consent from individual patients was not required.

The study population included adult patients (aged ≥40 years) with at least one recorded measurement of LDL-C in the ESPBI IS database between 1 January 2023 and 31 December 2023. The lower age limit was chosen because current clinical guidelines recommend lipid screening and treatment primarily from middle age onward, and CVD prevalence is low below this age, making LDL-C target evaluation less clinically relevant in younger adults. Measurements were extracted automatically by the SDA from semi-structured records in the observation field in the referral form (E027). The automated extraction relied on various pattern-matching rules, and the data provider estimates its accuracy at >95%. Patients were excluded if they were younger than 40 years or had a documented cancer diagnosis (International Classification of Diseases, 10th Revision [ICD-10] codes C00-C96). In cases of multiple LDL-C measurements during the study period, only the most recent value was used for the analysis.

The primary outcome was the proportion of patients achieving LDL-C concentration below 1.8 mmol/L, the target level recommended by the ESC guidelines for individuals at high cardiovascular risk [2].

For analysis, patients were classified into two groups—primary and secondary CVD prevention—based on ICD-10 diagnostic codes. Patients were assigned to the secondary prevention group if they had a documented prior cardiovascular event, i.e., ischemic heart disease (I25, I21), peripheral artery disease (I70), ischemic stroke (I63), or a history of cardiovascular procedures (Z95). Those without any of these cardiovascular events were classified as the primary prevention group. The secondary prevention group was further stratified into four subgroups based on specific diagnoses—ischemic stroke, atherosclerosis, other CVDs (I25, I21, Z95)—and a very high cardiovascular risk group, defined as patients having two or more of the aforementioned diagnoses.

The following lipid-lowering therapy (LLT) prescriptions were identified: statin monotherapy (Anatomical Therapeutic Chemical Classification System code C10AA), ezetimibe monotherapy (C10AX09), fixed-dose combination (FDC) of atorvastatin and ezetimibe (C10BA05), FDC of rosuvastatin and ezetimibe (C10BA06), FDC of statin with an antihypertensive agent (C10BX), fenobirate (C10AB05), omega-3-triglycerides (C10AX06), inclisiran (C10AX16), and PCSK9 inhibitors, including alirokumab (C10AX14) and evolocumab (C10AX13). Due to the low frequency of prescriptions for certain agents, LLTs were grouped for analysis as follows: (i) no LLT, (ii) statin monotherapy, (iii) FDC or free combination of statin and ezetimibe, (iv) FDC of statin and an antihypertensive agent, and (v) other lipid-modifying agents (i.e., ezetimibe monotherapy, PCSK9 inhibitors, inclisiran, fenofibrate, or omega-3-triglycerides).

The number of LLT prescriptions per year was categorized as follows: (i) no prescriptions, (ii) 1–3 prescriptions, and (iii) 4–12 prescriptions. (Note: In Lithuania, the maximum duration for a single prescription is three months; therefore, receiving 1–3 prescriptions in a year may indicate delayed treatment initiation, temporary interruption, or discontinuation within the year.)

The number of cardiologist consultations per year was categorized as (i) none, (ii) one consultation, and (iii) more than one consultation.

Data for the analysis were extracted from multiple components of the ESPBI IS database, including separate files for LDL-C concentrations, medical diagnoses, prescriptions and dispensations, and demographic data. These datasets were subsequently merged for analysis. Due to differences in data completeness across sources, some variables were missing for a subset of patients (e.g., sex information). Consequently, the total number of patients included in specific analyses varied depending on data availability for the variables of interest.

Several steps were taken to minimize bias: (1) selection bias was reduced by including all eligible patients in Lithuania meeting the inclusion criteria; (2) information bias was minimized through standardized, automated data extraction with >95% estimated accuracy; (3) misclassification bias was addressed by applying consistent diagnostic and treatment coding rules.

Descriptive statistics were used to summarize patient characteristics, LDL-C concentrations, LLT prescriptions, and cardiologist consultations. Qualitative variables were presented as frequencies and percentages, while quantitative variables were summarized using means and standard deviations. The proportion of patients achieving LDL-C targets was compared across subgroups using the chi-square test. Univariate binary logistic regression analyses were conducted to identify factors associated with achieving the LDL-C target level. Odds ratios (ORs) with 95% confidence intervals (CIs) were reported. All data transformations and descriptive statistics were carried out in the State Health Data Reuse Platform (implemented in original version of Palantir Foundry), using its Contour tool for data transformations. Other statistical analyses were performed using IBM SPSS Statistics version 29. Differences with a two-sided *p*-value <0.05 are considered statistically significant.

## 3. Results

A total of 396,835 patients aged ≥40 years with at least one recorded LDL-C measurement in 2023 and no documented cancer diagnosis were included in the analysis. Due to limitations in data merging, information on sex was missing for 91.58% of patients and on age for 91.6% of patients. Among those with available data, 16,138 were women and 17,263 were men; the mean (standard deviation) age was 66.91 (11.25) years. Most patients (94.58%) were classified as receiving primary CVD prevention. More than half of patients (56.35%) were not prescribed any LLT, and the vast majority of patients (81.18%) had no cardiologist consultations within the study period. Among those who received LLT, statin monotherapy was the most commonly prescribed treatment (Table 1).

Marked differences were observed in LLT use and cardiologist consultations between primary and secondary CVD prevention groups (Table 2). As expected, more patients in secondary prevention were prescribed statin therapy (as monotherapy or in combination with ezetimibe or an antihypertensive agent) and had more frequent LLT prescriptions. They also had significantly more cardiologist consultations, with over 40% of patients seeing a cardiologist at least twice a year.

The mean LDL-C concentration was 3.32 (1.14) mmol/L, and overall, only 8.1% of patients achieved an LDL-C of <1.8 mmol/L (Table 1). Table 3 presents the proportion of patients achieving this target level across subgroups. Target attainment was markedly higher among those receiving secondary CVD prevention compared to primary prevention (20.6% vs. 7.3%). In the secondary CVD prevention group, patients at very high cardiovascular risk had the highest rate of achieving an LDL-C level of <1.8 mmol/L (26.2%). The proportion of patients meeting the LDL-C target increased with more frequent cardiologist consultations and higher frequency of LLT prescriptions. Among LLT types, the highest target achievement was observed in patients treated with a combination of statin and ezetimibe (Table 3).

To further explore the factors associated with achieving LDL-C levels below 1.8 mmol/L, univariate logistic regression analysis was performed. It showed that achieving the LDL-C target was significantly (all *p* < 0.001) associated with being in the secondary CVD prevention group, having more frequent cardiologist consultations and more frequent LLT prescriptions (Table 3). Compared to patients not receiving LLT, the odds of achieving an LDL-C < 1.8 mmol/L were significantly higher in those receiving statin monotherapy (OR 3.153, 95% CI 3.069–3.240), statin in free or fixed combination with ezetimibe (OR 7.631, 95% CI 7.267–8.013), or statin in FDC with an antihypertensive agent (OR 3.945, 95% CI 3.803–4.092).

## 4. Discussion

In this nationwide retrospective analysis of health data, only 8.1% of patients aged ≥40 years achieved an LDL-C level of <1.8 mmol/L. LDL-C target achievement was markedly higher in the secondary CVD prevention group compared to the primary prevention group (20.6% vs. 7.3%), reflecting a more intensive treatment approach in individuals with established CVD. Recently published results from a single primary-healthcare center in Lithuania similarly reported LDL-C <1.8 mmol/L in only 4.6% of patients with dyslipidemia and in 23.8% of those with both dyslipidemia and CVD [18].

Despite the positive trends observed in dyslipidemia management over recent decades, it remains a major healthcare problem in Lithuania [18,19,20] and across Europe [21,22,23,24,25,26,27,28]. To achieve LDL-C goals, the 2019 ESC/EAS guidelines recommend sequential intensification of LLT, starting with statin up-titration, followed by the addition of ezetimibe, and subsequently incorporating a PCSK9 inhibitor [9]. Lipid-lowering therapy is most effective when started early, applied intensively, and continued long-term, i.e., reflecting the principles “the earlier, the better,” “the lower, the better,” and “the longer, the better” [17]. Such an approach significantly reduces the risk of cardiovascular events and mortality, as confirmed in numerous large studies and meta-analyses [29,30,31,32,33,34].

Nonetheless, suboptimal LLT use (i.e., only moderately intensive statin therapy and the underutilization of combination LLT) seems to be the key factor contributing to the low rate of patients achieving their LDL-C targets [17]. In our study, more than half of all patients were not prescribed any LLT, and fewer than 3% of patients received the combination therapy with statins and ezetimibe. These numbers are even lower than those reported in the SANTORINI study, where 22% of patients had no documented LLT and 24% received combination LLT [26], and the DA VINCI study, where 9% of patients used statins in combination with ezetimibe [22]. Many other observational studies have also shown that patients eligible for LLT are often undertreated [28,35,36,37,38]. Contributing factors include therapeutic inertia among clinicians [22], differing LLT prescribing practices between general practitioners and specialists [39,40], and limited reimbursement for ezetimibe. Clinicians also report concerns about cost-effectiveness, safety, patient adherence, and increased workload, as well as skepticism toward treating asymptomatic individuals and the complexity of cardiovascular risk assessment tools [41].

We categorized LLT into three groups: statin monotherapy, statin plus ezetimibe, and an FDC of statin with an antihypertensive agent. Due to the low number of ezetimibe prescriptions, patients receiving statins and ezetimibe, either as separate agents or in an FDC, were analyzed together. Consequently, the specific effects of FDC on these agents were not evaluated separately. Patients receiving statin–ezetimibe combination had the highest likelihood of achieving the LDL-C target (OR 7.631), highlighting the benefit of combining ezetimibe with statin, irrespective of statin intensity. This aligns with growing evidence that combining a low- or moderate-intensity statin with ezetimibe may offer better effectiveness and tolerability than using high-intensity statin therapy alone [15,16,42] and significantly reduces all-cause mortality, major adverse cardiovascular events, and stroke incidence [7].

Another barrier to achieving LDL-C targets is nonadherence to statin therapy, both in primary and secondary CVD prevention [43]. Adherence varies widely (18–80%), and discontinuation rates approach 50% in some settings [44,45,46,47]. We did not have specific data to assess patient adherence. A higher number of LLT prescriptions per year was associated with significantly better LDL-C control. However, the meaning of this finding remains unclear. While more frequent prescriptions may suggest more sustained or intensive treatment, this variable could also reflect other underlying factors not captured in our analysis. For example, a higher prescription count could result from a later initiation of LLT within the year, changes in treatment regimen, or more frequent prescription renewals due to patient preference. Given these uncertainties, this association should not be interpreted as a proxy of adherence or treatment persistence.

Patients in secondary prevention were more likely to achieve LDL-C goals in our study. This observation aligns with findings from other studies that reported better LDL-C control among secondary prevention patients compared to those in primary prevention [18,28]. Although the underlying reasons remain uncertain, potential contributors include closer clinical monitoring, more intensive LLT, timely treatment adjustments, and greater patient motivation following a cardiovascular event. In our study, more frequent consultations with cardiologists also appeared to support better LDL-C control. While causality cannot be established, specialist care may contribute through greater familiarity with guidelines, earlier treatment intensification, and more individualized management. However, published data on the differences in dyslipidemia management between general practitioners and specialists are limited and somewhat inconsistent. For example, an early US study reported that cardiologists were more proactive than general practitioners in treating hyperlipidemia after myocardial infarction, with their patients being more than twice as likely to receive LLT [39]. Similarly, another study observed markedly different prescribing patterns, with LLT initiated in fewer than 15% of coronary heart disease patients by family physicians, compared to 62.5% by internists and 34.7% by cardiologists [40]. In contrast, a recent Canadian study found no difference in LDL-C reduction or target achievement between patients managed by primary care physicians and specialists. Interestingly, that study noted a higher proportion of patients under specialist care were not receiving statins, although these specialists were more likely to prescribe guideline-recommended adjunct therapies such as ezetimibe or PCSK9 inhibitors [48]. Furthermore, a cross-sectional survey presenting identical clinical scenarios found that cardiologists tended to estimate cardiovascular risk lower than general practitioners and internists, while internists were more likely than them to initiate LLT [49]. These mixed findings indicate that further research is needed to understand how provider type and care structure affect LDL-C management outcomes.

This study has several strengths, including the use of comprehensive, real-world data from the national ESPBI IS database, enabling analysis across a large and diverse patient population with detailed laboratory, prescription, and diagnostic information.

However, several limitations should be acknowledged. First, we applied a uniform LDL-C target of 1.8 mmol/L to all patients, regardless of individual CVD risk. This was necessary because key variables required for cardiovascular risk stratification (e.g., smoking status, blood pressure, family history of CVD, diabetes duration and control) were not consistently available. Although this limitation precluded stratification by risk category as recommended in clinical guidelines, the use of a single conservative threshold minimized misclassification and ensured a consistent and pragmatic evaluation of LDL-C management across the study population. Second, we were not able to confirm whether prescribed LLTs were actually taken by patients. Third, the absence of data on age and sex for the majority of patients is a major limitation that prevented analysis of potential age- and sex-related differences in LDL-C management and may reduce the generalizability of our findings.

Our findings highlight substantial gaps in LDL-C target achievement and the underutilization of LLT. To improve cardiovascular outcomes, efforts should focus on earlier initiation and sustained intensification of LLT in line with current guideline recommendations. Increasing the use of combination therapy, particularly statin plus ezetimibe, may offer a pragmatic and effective strategy to help more patients reach LDL-C goals and reduce the burden of atherosclerotic CVD in Lithuania.

## 5. Conclusions

This nationwide analysis revealed that only 8.1% of patients achieved the LDL-C target of <1.8 mmol/L, with a low overall use of LLT. Target attainment was highest among patients in secondary prevention, those receiving statin–ezetimibe combination therapy, and those with more frequent cardiologist consultations. These findings highlight the need for broader implementation of intensive, guideline-recommended lipid-lowering strategies and may inform public health initiatives, clinical practice updates, and reimbursement policies aimed at improving LDL-C management in Lithuania.

## Figures and Tables

**Table 1:** Clinical characteristics of the study population.

Characteristics	
LDL-C concentration	
mean (SD), mmol/L	3.32 (1.14)
<3 mmol/L, *n* (%)	163,884 (41.3)
<1.8 mmol/L, *n* (%)	31,970 (8.1)
<1.4 mmol/L, *n* (%)	9980 (2.5)
Primary CVD prevention, *n* (%)	375,357 (94.58)
Secondary CVD prevention, *n* (%)	21,478 (5.41)
Ischemic stroke, *n* (%)	2165 (0.55)
Peripheral artery disease, *n* (%)	3735 (0.94)
Other CVDs, *n* (%)	14,134 (3.56)
Very high cardiovascular risk *, *n* (%)	1444 (0.36)
Number of cardiologist consultations, mean (SD)	2.02 (1.48)
Patients with no cardiologist consultations, *n* (%)	322,131 (81.18)
Lipid-lowering therapy, *n* (%)	
None	223,612 (56.35)
Statin monotherapy	124,188 (31.29)
Statin and ezetimibe combination	10,571 (2.66)
Statin and antihypertensive FDC	34,253 (8.63)
Other lipid-lowering agents **	4211 (1.06)

CVD, cardiovascular disease; LDL-C, low-density lipoprotein cholesterol; SD, standard deviation. * patients with two or more of cardiovascular events; ** ezetimibe monotherapy, PCSK9 inhibitors, fenofibrate, or omega-3-triglycerides.

**Table 2:** Lipid-lowering therapy and cardiologist consultations in primary and secondary cardiovascular disease prevention groups.

Characteristics	Primary CVD Prevention	Secondary CVD Prevention
Lipid-lowering therapy, n (%)		
None	223,612 (60.2)	0
Statin monotherapy	109,611 (29.5)	14,577 (69.4) *
Statin and ezetimibe combination	7682 (2.1)	2889 (13.8) *
Statin and antihypertensive FDC	30,717 (8.3)	3536 (16.8) *
Number of lipid-lowering agent prescriptions per year, n (%)		
None	223,612 (60.2)	0
1–3 prescriptions	104,938 (28.2)	11,823 (56.3) *
4–12 prescriptions	43,072 (11.6)	9179 (43.7) *
Number of cardiologist consultations per year, n (%)		
None	316,006 (84.2)	6125 (28.5) *
1 consultation	31,552 (8.4)	5740 (26.7) *
≥2 consultations	27,799 (7.4)	9613 (44.8) *

* *p* < 0.001 for overall comparison across all categories of a variable (chi-square test).

**Table 3:** Univariate logistic regression analysis of factors associated with achieving LDL-C <1.8 mmol/L.

Factors	Patients with LDL-C <1.8 mmol/L, n (%)	*p* Value *	OR (95% CI)	*p* Value **
CVD prevention group				
Primary prevention (reference)	27,545 (7.3)	<0.001	-	
Secondary prevention	4425 (20.6)		3.277 (3.163–3.394)	<0.001
Ischemic stroke	300 (16.2)		2.442 (2.157–2.765) ^†^	<0.001
Peripheral artery disease	499 (15.5)		2.317 (2.104–2.551) ^†^	<0.001
Other CVDs	2534 (20.7)		3.299 (3.152–3.452) ^†^	<0.001
Very high cardiovascular risk ***	1092 (26.2)		4.474 (4.171–4.799) ^†^	<0.001
Number of cardiologist consultations per year				
None (reference)	19,340 (6.0)	<0.001	-	
1 consultation	5659 (15.2)		2.801 (2.713–2.891)	<0.001
≥2 consultations	6971 (18.6)		3.585 (3.480–3.694)	<0.001
Lipid-lowering therapy				
None (reference)	9207 (4.1)	<0.001	-	
Statin monotherapy	14,811 (11.9)		3.153 (3.069–3.240)	<0.001
Statin and ezetimibe combination	2609 (24.7)		7.631 (7.267–8.013)	<0.001
Statin and antihypertensive FDC	4962 (14.5)		3.945 (3.803–4.092)	<0.001
Number of lipid-lowering agent prescriptions per year				
1–3 prescriptions (reference)	9759 (8.4)	<0.001	-	
4–12 prescriptions	12,623 (24.2)		3.493 (3.393–3.595)	<0.001

CI, confidence interval; CV, cardiovascular; CVD, cardiovascular disease; FDC, fixed-dose combination; LLT, lipid-lowering therapy; OR, odds ratio. * *p* value for overall comparison across all categories of a variable (chi-square test), ** *p* value from logistic regression, *** patients with two or more cardiovascular events, ^†^ the reference group is “Primary prevention”.

## Data Availability

Data are available by applying to the State Data Agency following the Law for Health Data Reuse, https://duomenys.stat.gov.lt/health-data/ (accessed on 12 March 2024).

## Abbreviations

The following abbreviations are used in this manuscript:

CI	Confidence interval
CVD	Cardiovascular disease
EAS	European Atherosclerosis Society
ESC	European Society of Cardiology
ESPBI IS	Electronic Health Services and Cooperation Infrastructure Information System
FDC	Fixed-dose combination
ICD-10	International Classification of Diseases, 10th Revision
LDL-C	Low-density lipoprotein cholesterol
LLT	Lipid-lowering therapy
OR	Odds ratio
PCSK9	Proprotein convertase subtilisin/kexin type 9
SD	Standard deviation
SDA	State Data Agency
